# Case report: Multimodal neoadjuvant and adjuvant chemotherapy for hepatic undifferentiated embryonal sarcoma in a young adult

**DOI:** 10.3389/fonc.2022.1004108

**Published:** 2022-11-18

**Authors:** Rosemary Vergara, Sarah Khalil, Gitonga Munene

**Affiliations:** ^1^ School of Medicine, Western Michigan University Homer Stryker MD School of Medicine, Kalamazoo, MI, United States; ^2^ Department of Surgery, Western Michigan University Homer Stryker MD School of Medicine, Kalamazoo, MI, United States; ^3^ West Michigan Cancer Center, Kalamazoo, MI, United States

**Keywords:** embryonal sarcoma of adult liver, hepatectomy, neoadjuvant chemotherapy, undifferentiated embryonal hepatic sarcoma, UESL

## Abstract

Hepatic undifferentiated embryonal sarcoma of the liver (UESL) is a rare hepatic malignancy found more commonly in pediatric patients. It has been associated with poor outcomes in adults and the role and timing of systemic therapy is unclear. There have been very few case reports detailing combination neoadjuvant and adjuvant chemotherapy use for hepatic undifferentiated embryonal sarcoma in adults. In this report, a 22-year-old male admitted with right upper quadrant pain was diagnosed with a 20 x 10 x 10 cm well-circumscribed, highly vascularized hepatic mass in the entirety of the left lobe. Biopsy confirmed the diagnosis of UESL. PET/CT showed no evidence of metastatic disease, and he received four cycles of Doxorubicin and Ifosfamide with demonstrated reduction in size and decrease in PET avidity. He underwent left hepatectomy with periportal lymphadenectomy, cholecystectomy, and partial gastrectomy with negative margins and received adjuvant Doxorubicin, Ifosfamide and Mesna. At 48 months, the patient was alive without evidence of disease. We hereby emphasize the potential advantages of combination chemotherapy and surgical resection in the management of UESL in adults.

## Background

First described in 1978, undifferentiated embryonal sarcoma (UESL) of the liver is a rare mesenchymal tumor, almost exclusively found in pediatric age patients (1–3). The average age of presentation is approximately 6 to 10 years of age with over 88% of cases occurring in patients 15 years of age or younger (1, 4). Diagnosis can be difficult, as there are shared clinical and radiological findings with other hepatic tumors. The pathogenesis is not well-described but is possibly associated with mesenchymal hamartoma as both tumors have been associated with similar cytogenetic abnormalities (3).

Accounting for less than 1% of all primary liver neoplasms in adults, an optimal treatment regimen for UESL is still being determined (5). Surgical resection of the tumor was previously the mainstay of treatment for UESL (3). Recently, several studies have indicated multimodal therapy with surgery, radiation, and chemotherapy can significantly improve patient prognosis (3). Despite aggressive treatment, a diagnosis of UESL carries an overall 5-year survival rate estimated between 65% to 72% (6, 7). Adults have been reported to have a worse prognosis than in pediatric patients with a reported survival of 48.2% (7). Given the high rates of regional and local recurrence, a strategy employing early systemic therapy may have the potential to improve outcomes. There is limited data on the efficacy and feasibility of multimodal therapy in this rare tumor type. This report outlines the successful treatment of a male adult patient with UESL treated with neoadjuvant and adjuvant chemotherapy in combination with surgical resection.

## Case presentation

A 22-year-old male presented with one month of decreased appetite and sharp, intermittent epigastric pain radiating to the right upper quadrant. The patient did have a history of anemia but was otherwise healthy with no significant family history. Physical exam revealed abdominal distension, with visible protrusion in the LUQ, and no tenderness. Laboratory tests revealed anemia and neutropenia with elevated alkaline phosphatase, lipase, lactate dehydrogenase and bilirubin (WBC 2.1, normal 4 -11 g/L; Hemoglobin 8.0, normal 13.5 - 17.5 g/dL; Alkaline phosphatase 179, normal 39 - 117; lipase 1140, normal 16 - 63 U/L; LDH 569, normal 94 - 250 U/L, Bilirubin 1.0, normal < 0.3 mg/dL). Alpha Fetoprotein, AST and ALT levels were normal. Hepatitis serologies were negative.

**Figure 1:**
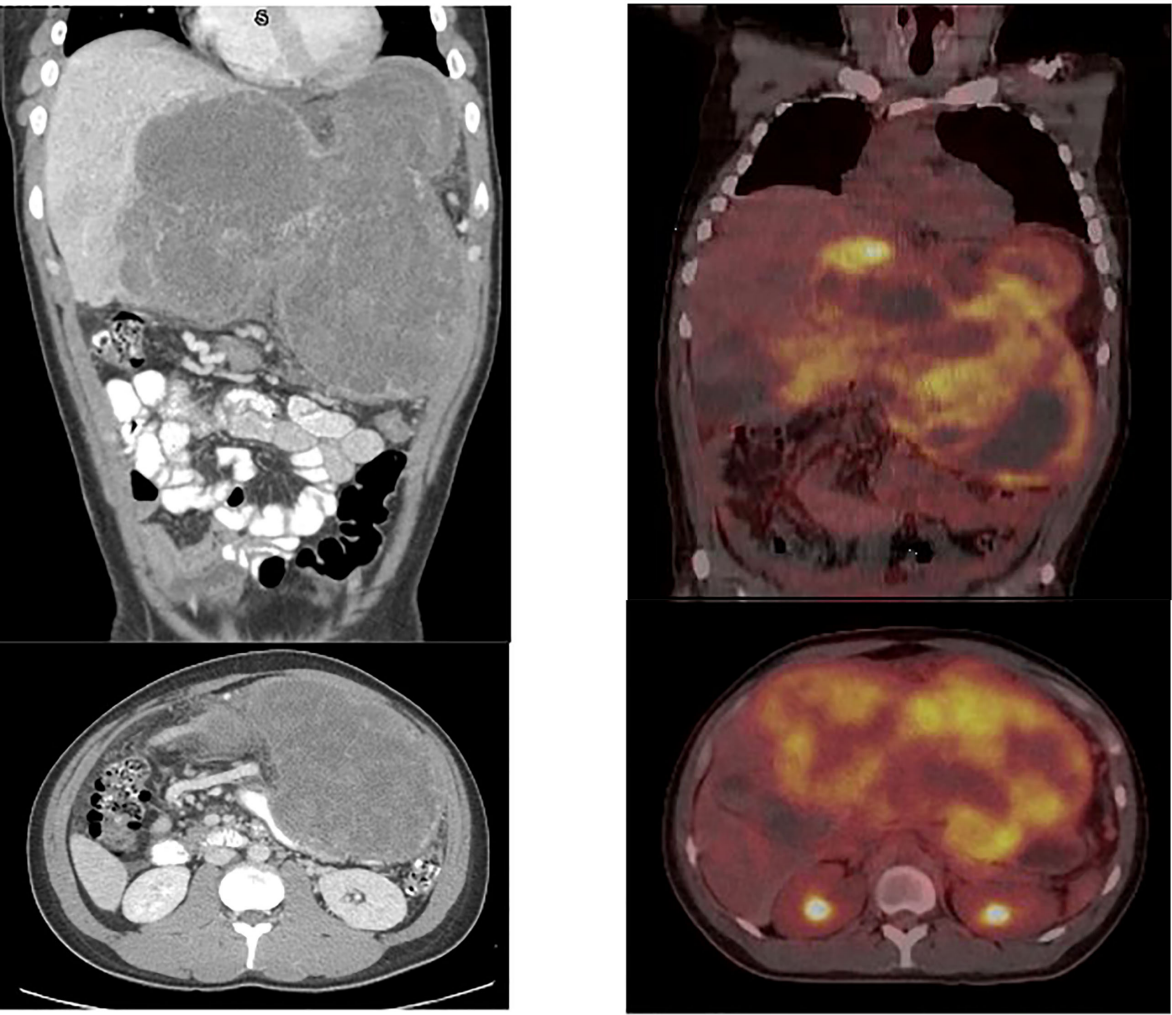
Initial CT scan demonstrating UESL. 20 x 10 x 10 cm well-circumscribed, solid mass of the entirety of the left lobe of the liver with relative sparing of the right lobe. Initial PET/CT demonstrating large hepatic mass measuring 25 x 13 cm with maximal SUV 11.5 as well as areas of necrosis, demonstrated by lack of FDG avidity.

**Figure 2:**
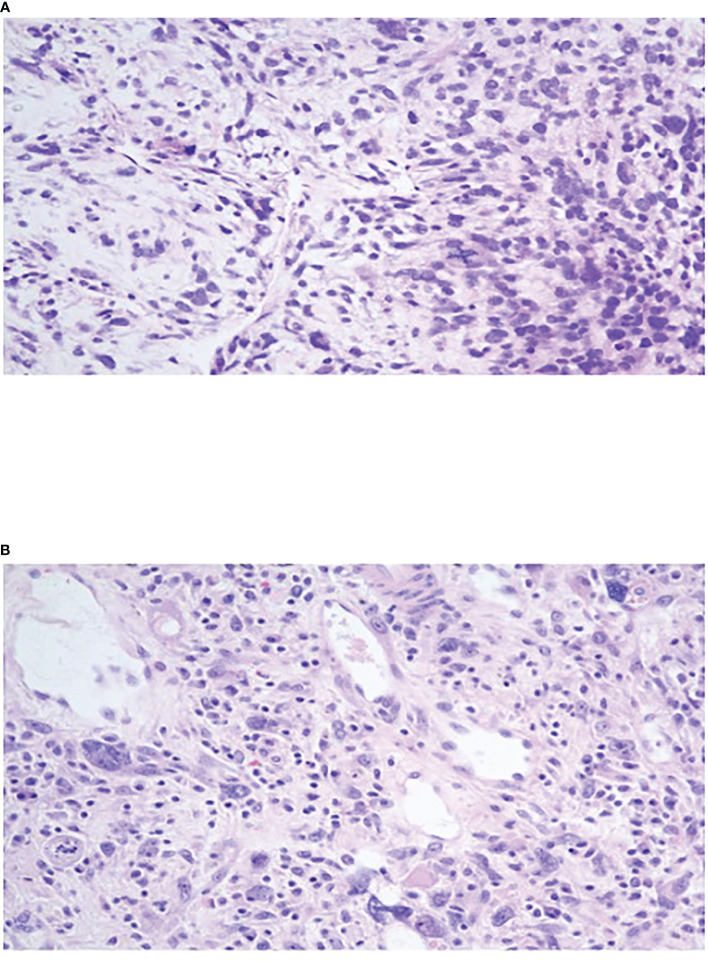
**(A)** Oval to stellate tumor cells with ill-defined cell borders loosely distributed in myxoid stroma with marked nuclear pleomorphism, hyperchromasia and brisk mitotic activity. **(B)** Tumor cells within a fibroinflammatory background showing marked nuclear pleomorphism, hyperchromasia, and frequent multinucleated and bizarre giant cells. Cytoplasmic and extracellular eosinophilic globules noted.

Abdominal computed tomography (CT) revealed a solitary 20 x 10 x 10 cm well-circumscribed, highly vascularized hepatic mass in the entirety of the left lobe with relative sparing of the right lobe (**Figure 1**). Positron emission tomography (PET) Scan redemonstrated this hepatic mass demonstrating intense FDG uptake of SUV 11.5 and areas of necrosis (**Figure 1**). There was no evidence of metastatic disease and no evidence of invasion into surrounding structures. Ultrasound guided core needle biopsy identified a malignant spindle cell neoplasm consistent with diffuse embryonal sarcoma (**Figure 2**). Biopsy results combined with imaging were diagnostic of UESL.

**Figure 3:**
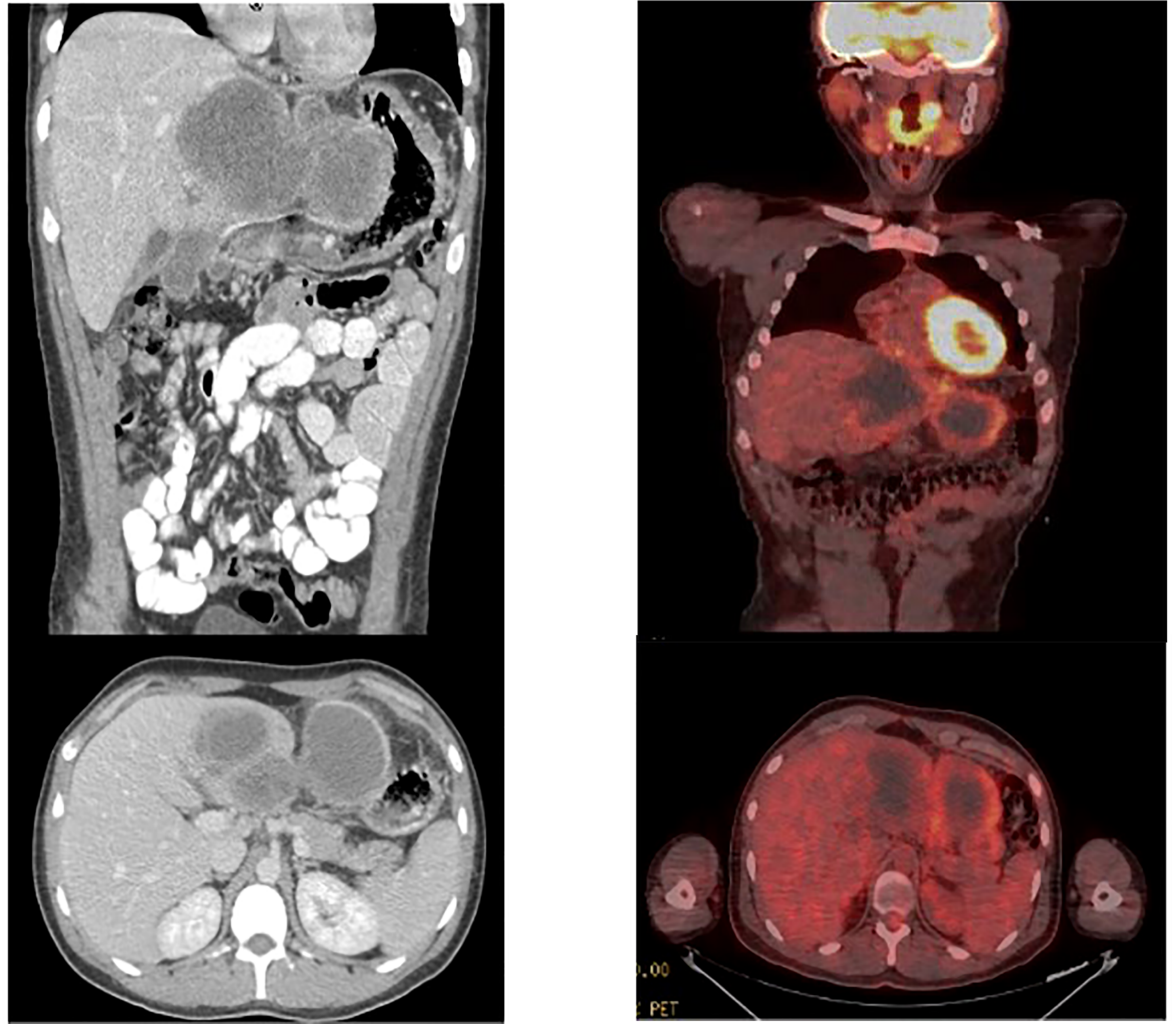
CT scan after completion of neodjuvant chemotherapy. Significant improvement was visualized with a decrease in lesion size to 15 x 7.7 x 8.1 cm. PET scan after completion of neodjuvant chemotherapy demonstrating reduction in size of mass to 14.5 x 6.8 x 8.9 cm as well as decreased FDG avidty of SUV 5.4.

Patient was treated with neoadjuvant chemotherapy modeled after protocol ARST 1321 Regimen B by the Children’s Oncology Group. Preoperative chemotherapy consisted of Ifosfamide (2.5g/m^2) per dose intravenously on days 1 to 3) with MESNA and doxorubicin (37.5 mg/m^2 per dose intravenously on days 1 to 2) at 3-week intervals for 4 cycles. Beyond generalized malaise, the patient did not experience any adverse side effects to chemotherapy. Repeat CT scan indicated a decrease in tumor size to 15.2 x 7.7 x 8.1 cm. Repeat PET scan corroborated the decrease in tumor size and indicated a significant decrease in FDG uptake, with a maximum SUV of 5.4 (**Figure 3**).

Intraoperatively, the hepatic mass of the left lobe was identified with significant omental and gastric adhesions. The tumor was also invading the medial gallbladder wall. Intraoperative ultrasound was used to delineate intraparenchymal tumor borders and ultimately the patient underwent left hepatectomy with periportal lymphadenectomy, cholecystectomy, and partial gastrectomy. Surgical margin was negative defined as greater than 1mm. The patient recovered with no surgical complications. Postoperatively, the patient underwent an additional two cycles of adjuvant Doxorubicin, Ifosfamide and Mesna. 3 weeks after surgery the patient received 2 cycles of Doxorubicin/Ifosfamide and one of Doxorubicin only at 3 week intervals, with or without Pazopanib, completing all therapy at week 25 (cumulative doses: Ifosfamide 45 g/m^2, Doxorubicin 375 mg/m^2).

For surveillance the patient underwent CT or MRI of the abdomen and pelvis plus a chest X-ray or CT. Surveillance protocol included CT scan every three months for the first year, every four months the second year, every six months through the third year and then once yearly beyond three years. The patient has since been noted to be in good health with no evidence of local or distant recurrence at present, 4 years post-resection.

## Discussion

At 2 to 15% of cases, UESL is the third most common hepatic malignancy in pediatric populations after hepatoblastoma and hepatocellular carcinoma (1, 4, 8). It is estimated that only 47 cases of UESL in patients older than 15 years old have been reported in the literature from 1978 to 2007 (9). A more recent review from 1973-2019 reported less than 90 adult UESL cases and a recent NCDB study only identified 41 adult patients (6, 7). Patients with UESL can present with nonspecific symptoms including abdominal pain, fever, weight loss diarrhea or vomiting (1, 4, 9–11). Laboratory evaluation may reveal leukocytosis and anemia; however no specific serum markers identify UESL (1, 3). The differential diagnosis of UESL includes abscess, mesenchymal hamartoma, hydatid cyst, hepatocellular carcinoma, hepatoblastoma, biliary tract rhabdomyosarcoma, cystic metastasis, angiolipoma, leiomyosarcoma, liposarcoma, epithelioid hemangioendothelioma, and malignant melanoma (1, 3, 8). UESL does share chromosomal abnormalities seen with mesenchymal hamartoma, with some suggesting malignant transformation of mesenchymal hamartoma to develop UESL; abnormalities associated with 19q13.4 including balanced translocations t(11;19)(q13;q13.4) and t(15;19)(q15;q13.4) (1, 3, 12).

UESL can have a differential appearance on imaging. Ultrasound demonstrates a solid hepatic mass, generally isoechoic to normal liver, with anechoic areas corresponding to areas of tumor necrosis or degeneration (1, 13). CT demonstrates a well-defined hypodense mass with internal septations, almost cystic in appearance (1, 13). With contrast administration the tumor does progressively enhance (1, 13). MRI demonstrates low signal intensity on T1 weighted images and high signal intensity on T2 weighted images, again with enhancement on contrast administration (1, 13). Due to its differential appearance, image-guided biopsy is the mainstay of diagnosis, however cases have been reported of tumor rupture after biopsy (14). Rupture prior to complete surgical resection can be a consideration for treatment of residual disease and higher risk of recurrence (2, 4). The prognosis of UESL with rupture into the peritoneal cavity is less favorable in comparison to cancerous counterparts including hepatocellular carcinoma secondary to factors including increased risk of systemic metastases (15).

Histopathology demonstrates that UESL is composed of undifferentiated spindle cells with mitotic figures and myxoid stroma (9, 16). The high water content of the myxoid stroma is thought to be the reason for the differential appearance of UESL on imaging (1, 13). UESL tumors also show characteristic intracellular hyaline globules and anaplasia on a mesenchymal background (8, 17). These tumors tend to have a pseudocapsule made of compressed hepatic parenchyma, leading to a well circumscribed hepatic mass (9).

Prognosis for UESL has historically been considered dismal with reported 5-year overall survival rate of approximately 65% in all patients with worse outcomes reported in adults (6, 18). Factors that have been associated with improved survival are margin negative resection, receipt of chemotherapy and childhood. Treatment options for UESL include surgery, chemotherapy, and liver transplantation (1, 19–21). One retrospective study of UESL patients from 1975 to 2015 using the SEER database showed while 84% of patients underwent surgery, only 65% underwent chemotherapy and 9% underwent radiation therapy (18). While surgical resection of the primary tumor was previously the mainstay of treatment, recent studies have shown success with use of adjuvant chemotherapy and neoadjuvant chemotherapy, particularly in initially unresectable cases (3). Tumors are generally considered unresectable due to size, amount of liver involvement, and invasion into surrounding structures (14). Given the poorer outcome in adults and the propensity for early local and distant metastases a strategy employing combined neoadjuvant and adjuvant chemotherapy may result in improved outcomes. A recent review of NCDB patients with UESL from 2004 – 2015 showed that 92.7% of pediatric patients received chemotherapy as opposed to 65.9% of adults (7). Only 1 out of 41 adult patients receiving both surgery and chemotherapy also received neoadjuvant therapy as opposed to adjuvant therapy (7).

As shown in one study, 12 of 17 children with UESL treated with a multimodal approach between 1979 and 1995 were alive at 2.4 to 20 years follow-up (4). A separate study of five pediatric patients treated with resection followed by adjuvant chemotherapy demonstrated no disease recurrence at a median of 53 months post-treatment (22). A third study of five pediatric patients ages 10 to 19 were also treated with multimodal therapy, including orthotopic liver transplantation in unresectable cases, demonstrating 100% survival at 21 to 68 months of follow-up with instances of recurrence (23). Combinations of Vincristine, Cyclophosphamide, Dactinomycin, Doxorubicin, Etoposide and Ifosfamide are associated with longer survival and disease-free prognosis (4, 8, 10, 24). Chemotherapy for UESL is modeled after established regimens for pediatric rhabdomyosarcoma or Ewing sarcoma without cisplatin (8, 17, 25). UESL metastases have been reported in up to 15% of pediatric patients and are mainly seen during primary diagnosis in which sites most commonly include lungs, adrenal glands, peritoneum and extension into the heart (23, 26, 27). Available data regarding metastatic disease in UESL is limited but is a critical consideration for future optimization of multimodal treatment options.

A review of multimodal treatment regimens for UESL are outlined in **Table 1**. Also included are some cases of patients undergoing surgery alone with no chemotherapy, highlighting the improved success with neoadjuvant and adjuvant treatment strategies. Given the reported improvements associated with chemotherapy in pediatric patients, we elected to treat our patient with multimodal chemotherapy and surgical resection. The intended goal of this approach in our patient was decrease tumor size to enable resection, start early systemic therapy for a disease with a propensity for early metastases, and to assess tumor response *in vivo*. These advantages have been reported for other cancers such as gastric, gastroesophageal and pancreatic cancers (34, 35). Our patient thus far appears to have achieved some of the intended benefits of combination chemotherapy as well as disease free survival of 48 months. In conclusion, we believe this approach is well suited for this cancer given its underlying biology i.e. a propensity for early local and distant recurrence, and therefore warrants further study. This case study adds to the growing body of literature favoring combination of combined neoadjuvant and adjuvant chemotherapy with hepatic resection in treatment of UESL.

**Table 1:** Cases of multimodal treatment of UES in adults.

Reference	Year	Age/Sex	Tumor(s)	Treatment	Neoadjuvant Chemotherapy	Adjuvant Chemotherapy	Recurrence	Follow up (Months)
Present	2021	22 M	20 x 10 x 10 cm	Left hepatectomy.	DOX IFO MES	DOX IFO MES	None	48
Yu (19)	2021	69 F	16 cm	Surgical resection. RFA	None	None	6 months,	N/A
Pandit (20)	2019	29 F	15 x 12 cm	Surgical resection.	None	None	Yes at 4 months.	8; deceased.
Pandit (15)	2019	34 M	16 x 14 cm	Laparotomy.	None	None	Tumor progression at 6 months.	6
Pinamonti (28)	2018	60 F	23 x 15 x 12 cm	hepatectomy	None	ACT CYC VIN	None	30
Sanchez-Morales (21)	2018	53 F	Hypodense mass in the right lobe with satellite lesions.	Palliative radiotherapy and intravenous analgesia.	None	None	None	Deceased
Sanchez-Morales (21)	2018	41 F	20 x 16 cm mass in the right lobe.	Right hepatectomy.	None	Post-recurrence DOX ISO Scheduled DOCE GEM	24 months.	60
Khan (29)	2017	21 M	12.3 x 9.8 x 8.3 cm and 13.6 x 9.8 x 9.8 cm.	No tumor resection; unresectable. Orthotopic liver transplant.	DOX IFO	N/A	None	18
Giakoustidis (30)	2016	30 M	Voluminous mass in the right lobe.	Right portal vein embolization. Right trisegmentectomy.	None	CIS CYC DOX	Yes at 12 months.	28; deceased.
Zanwar (31)	2016	25 M	Hypodense mass in the right lobe.	Right hepatectomy.	ADRI IFO MES VIN	ADRI IFO MES VIN	None	24
Kim (32)	2011	47 F	12 x 10 cm well-demarcated mass in the left segment.	Left lateral sectionectomy. Radiotherapy.	DOX DZN IFO MES	None	Yes at 24 months, metastatic lesion treated with radiotherapy.	48
Noguchi (5)	2011	27 F	21 x 19 x 14 cm mass in the right liver lobe.	Right trisegmentectomy. Radiation therapy. Peripheral blood stem cell transplantation.	None	First Course ACT ADRI CIS CYC VIN Second Course CAB CYC ETP RAN	None	60
Ma (33)	2008	61 F	12 x 9 x 8 cm mass to the right lobe.	Right hepatic lobectomy. Cholecystectomy.	None	None	None.	8; deceased.
Pachera (9)	2008	22 F	19 x 14 x 11 cm mass.	Right portal vein embolization. Right trisectionectomy with extrahepatic bile duct resection.	None	ACT CYC VIN	None	14
Almogy (16)	2005	21 F	15 x 15 cm mass confined to the right lobe. Second smaller lesion in the left lateral segment.	Hepatic trisectionectomy	None prior to first resection DOX IFO MES Prior to resection of secondary tumor	DOX IFO MES	None	71

Adriamycin (Adri), Actinomycin D (Act), Carboplatin (Cab), Cisplatin (Cis), Cyclophosphamide (Cyc), Dacarbazine (Dzn), Docetaxel (Doce), Doxorubicin (Dox), Etoposide (Etp), Gemcitabine (GEM), Ifosfamide (Ifo), Isophosphamide (ISO), Mesna (Mes), Pembrolizumab (PMB), Ranimustine (Ran), Vincristine (Vin).

## Data availability statement

The raw data supporting the conclusions of this article will be made available by the authors, without undue reservation.

## Ethics statement

This case report is exempt from IRB approval. Patient did provide consent to publication of this manuscript.

## Author contributions

RV and SK completed review of the literature, acquisition of data, drafting and completion of the manuscript. SK and GM participated in the critical review of the paper. All authors contributed to the article and approved the submitted version.

## Funding

Funding for publication was obtained from the Western Michigan University Homer Stryker MD School of Medicine Department of Surgery research fund.

## Acknowledgments

We would like to thank Bronson Methodist Hospital, West Michigan Cancer Center, and Western Michigan University for allowing us to conduct this project.

## Conflict of interest

The authors declare that the research was conducted in the absence of any commercial or financial relationships that could be construed as a potential conflict of interest.

## Publisher’s note

All claims expressed in this article are solely those of the authors and do not necessarily represent those of their affiliated organizations, or those of the publisher, the editors and the reviewers. Any product that may be evaluated in this article, or claim that may be made by its manufacturer, is not guaranteed or endorsed by the publisher.

## Abbreviations

ALT, Alanine aminotransferase; AST, Aspartate aminotransferase; CT, Computed tomography; FDG, F-18-Deoxyglucose or Fluorodeoxyglucose; NCDB, National Cancer Database; PET, Positron emission tomography; SEER, Surveillance, Epidemiology and End Results database; SUV, Standard uptake value; UESL, Undifferentiated embryonic sarcoma.
